# Acquiring evidence-based medicine and research skills in the undergraduate medical curriculum: three different didactical formats compared

**DOI:** 10.1007/s40037-014-0143-y

**Published:** 2014-11-14

**Authors:** M. Zee, M. de Boer, A. D. C. Jaarsma

**Affiliations:** 1 Center for Evidence-Based Education, Academic Medical Center, University of Amsterdam, Amsterdam, the Netherlands; 2 Research Institute of Child Development and Education, University of Amsterdam, Nieuwe Achtergracht 127, 1018 WS Amsterdam, the Netherlands; 3 Center for Research and Innovation in Medical Education, University Medical Center Groningen, Groningen, the Netherlands

**Keywords:** Undergraduate research project, EBM skill acquisition, Medical education, Multivariate differences

## Abstract

Medical schools have recently witnessed a call for authentic research activities that equip students with the skills required for evidence-based medicine (EBM) and research. Because it is not always possible to make such activities available as a part of the curriculum, evaluating the effectiveness of the various choices of traditional and authentic EBM and research skills courses is essential. This study’s purpose was to evaluate students’ perceived EBM and research skill acquisition in three different courses in a Dutch medical school. Self-reported surveys were conducted among 163 Dutch medical undergraduates who participated in an undergraduate research project, a basic EBM skills elective, or a traditional lecture-based skills course. MANCOVA was employed to test for group differences in perceived skill acquisition. Students who finished their research project perceived themselves as more experienced in writing and information retrieval skills than students who participated in the lecture-based course or basic skills elective. Students in the lecture-based course identified themselves as being the most experienced in critical judgment. No group differences were found for overall gains. Authentic research activities may have benefits over traditional lecture-based courses in the undergraduate medical curriculum, especially in terms of equipping students with writing and information retrieval skills.

Undergraduate medical curricula have recently witnessed an unparalleled call for active, student-centered learning activities that equip students with skills required for evidence-based research and practice [1, 2]. This call has resonated far and wide with a plethora of research-based courses that have been provided in an effort to instil the evidence-based medicine (EBM) and research skills in which medical students ought to be competent [3–6]. Traditionally, such courses have involved specialized, teacher-centred lectures or small-group sessions in which medical students are taught to apply introductory EBM principles, such as searching and critically appraising research evidence [7, 8]. As yet, however, curriculum design experts have increasingly turned to more *authentic* alternatives to foster skills for implementing EBM and research. These include, among others, (extracurricular) research electives, summer studentship programmes, and undergraduate theses [2, 9–11].

Consensus has emerged from several sources that authentic research courses may be an indispensable step for medical students to acquire EBM skills, and to successfully exercise evidence-based practice in clinical settings [12–14]. Indeed, there is empirical evidence to suggest that having research experience as a medical student is associated with various research capacities, including problem-solving skills, information retrieval skills, critical appraisal skills, and data collection skills [12, 13, 15–17]. Moreover, undergraduate research experience appears to be likely to help medical students to get better job and continuing education opportunities [9, 12, 18].

Despite the promising potential of authentic research projects, there are few, if any, studies that have directly compared its effectiveness with more traditional courses that aim to foster EBM skill acquisition. Because medical schools do not always have the time, space, or resources to make meaningful research activities available as a part of the core curriculum, assessing the relative effectiveness of the various choices of traditional and authentic skill courses is essential. Therefore, the present study sets out to compare students’ perceived EBM and research skill acquisition in three different skill courses in a Dutch medical school.

## Method

### Context

The curriculum of the Academic Medical Center (AMC), Amsterdam, is based on a two-cycle system beginning with an undergraduate Bachelor period and followed by a graduate Master phase. In the undergraduate curriculum, there are three courses developed to foster medical students’ EBM skill acquisition. The first is a traditional, lecture-oriented course of seven weeks in the second year of medical school. This course, which was implemented in the autumn of 2012, addresses topics such as critical appraisal, epidemiology, research designs, and information retrieval. In addition to this traditional course, second-year students have the possibility to do a basic skills elective, during which they accompany a faculty member for four fulltime weeks on an ongoing research project. Students’ EBM and research skills are assessed by a short research report, which they have to hand in before the course finishes.

Since 2011, third-year students have to participate in an undergraduate research project, which is an obligatory course with a duration of one academic year. It requires students to select a topic for independent research and to complete either a literature review, or a clinical project under the supervision of a staff member of the university. In contrast to the basic skills elective, students are expected to spend a large amount of independent study time on their projects. Moreover, students are offered weekly seminars over a period of three months. During these voluntary seminars, students learn to search for literature, to write scientifically, to critically appraise the literature, and to present their findings. At the end of the process, students have to demonstrate their degree of topic mastery in a written thesis and a presentation.

### Participants

Participants in this study were students who took part in the undergraduate research project (group 1), the basic skills elective (group 2), or the lecture-based course (group 3), all aimed to foster their EBM skill acquisition. The first group comprised 42 participants, and the last two comparison groups 59 and 62 participants, respectively. Students self-selected the condition in which they participated by registering for either the undergraduate research project or the (elective) skills courses in year 2. None of the third-year students had followed the basic skills elective or the traditional lecture-based course in their second year of medical school. Moreover, students who took part in the basic skills elective had not yet participated in the lecture-based skills course, since this course was newly implemented in the autumn of 2012. The demographic characteristics for each of the three student groups are summarized in Table 1. The age, grade point average (GPA), and distribution of gender in the groups appeared to be largely consistent with the general medical student population at the AMC.

**Table 1:** Demographic characteristics per comparison group

	Group 1 (N = 42)		Group 2 (N = 59)		Group 3 (N = 62)	
	M	SD	M	SD	M	SD
Gender						
Male	16	–	18	–	48	–
Female	26	–	41	–	14	–
Age	22.29	2.49	23.15	2.41	21.31	2.02
GPA	7.12	0.87	6.91	0.64	7.03	0.68

Students’ age ranges from 19.0 to 33.0. Students’ grade point average (GPA) is displayed on a scale from 1 to 10

*M* mean, *SD* standard deviation

### Procedure

During the autumn of 2012, all second- and third-year undergraduates at the AMC were asked by email to fill out digital self-report questionnaires about their EBM skill acquisition. A total of 163 students returned completed questionnaires, resulting in an overall response rate of 23.29 %. As the questionnaires used in this study were part of the AMC’s regular course evaluation cycle, ethical approval was not deemed necessary for this study. However, appropriate ethical principles and scientific practice were followed in accordance with the declaration of Helsinki. Prior to participation, informed consent was obtained from the students by providing them with a written account of the study’s purposes, and a permission form that could be signed by clicking on an ‘accept button’. All students participated on a voluntary basis and could opt out of the study at any moment. Any information that could be traced to students’ identity was removed from the data.

### Measures

#### EBM skills

Medical students’ experience in EBM and research was measured by a slightly adapted version of the College Student Experiences Questionnaire (CSEQ; see Appendix). This is a self-report questionnaire that can be used to estimate the effectiveness of a curriculum or its separate components in advancing student outcomes [19]. Previous investigators [20] have not only reported satisfactory reliability and validity for this instrument, but also underscored its potential for evaluating student activities that are well related to objective measures of achievement [21, 22].

Originally, the CSEQ consisted of 166 items, divided into several (sub)scales, which focus on the amount of time and effort students spend on learning activities (Activities items), students’ perceptions of the learning environment (Environment items), or students’ development of overall gains (Estimate of gains items). Most items were rated on a five-point Likert-type scale, ranging from 1 (never) to 5 (very often). For the purposes of the present study, use was made of a selection of the subscales, which are presented below. The Cronbach’s alphas of the scales were satisfactory, ranging between 0.71 and 0.90.

##### Student characteristics

Students’ background features (age, gender, and GPA, encompassing students’ clinical knowledge, abilities, and professional behaviour) were derived from individual CSEQ items about background information. Two additional dichotomous items, indicating whether students were second- or third-year undergraduates, and whether they participated in the undergraduate research project or the elective research course, were included to assign students into the three groups.

##### Information retrieval skills

Students’ information retrieval skills were represented by the library experiences scale, which measured the amount of time and energy students devote to retrieving information from the library, and reading study material. The eight items that made up this scale include statements such as ‘used an index or database (computer, card catalogue, etc.) to find material on some topic’.

##### Critical judgment

Students’ critical judgment skills were characterized by six items about exploring research topics and information in conversations. An example item of this scale is ‘explored different ways of thinking about the topic’.

##### Writing skills

Students rated seven items concerning their experiences with respect to writing during college. This writing skills scale was represented by students’ mean response to relevant items, and included items such as ‘thought about grammar, sentence structure, word choice, and sequences of ideas as you were writing’.

##### Statistical skills

The time and effort students spent on scientific and quantitative skills during their study were measured by the scientific and quantitative experiences scale. This ten-item measure comprised items such as ‘compared the scientific method with other methods for gaining knowledge and understanding’ and ‘practised to improve your skill in using a piece of laboratory equipment’.

##### Overall gains

Students’ EBM and research skill development was represented by 23 estimate of gains items, which gauge students’ opinions about the progress they have made in EBM and research skills. Questions in this subscale have often been linked to other performance-related measures, such as achievement tests [22], and show some links with the CanMEDS role of scholar. Examples of items of this scale, which was measured by the mean response to the items, were ‘putting ideas together, seeing relationships, similarities and differences between ideas’, and ‘presenting ideas effectively when speaking to others’.

### Study design and data analysis

A post-only non-experimental design was employed to compare students’ perceived EBM skill acquisition in the three skills courses. Unfortunately, random selection and assignment of students to the groups was not feasible, as students self-selected the condition in which they participated by registering for either the undergraduate research project or the (elective) EBM skills courses in year 2. To guard against inflated type-I error, differences in mean reported skill acquisition were analyzed using multivariate analysis of covariance (MANCOVA). For this analysis, SPSS MANOVA with the sequential adjustment for non-orthogonality was utilized. Five dependent variables were used to assess students’ EBM skill acquisition: writing skills, information retrieval skills, statistical skills, critical judgment skills, and perceived overall gains. Age, gender, and GPA were used as covariates to control for initial differences, and to provide statistical matching.

After multivariate statistical significance was detected, analyses proceeded with univariate and Roy-Bargmann stepdown *F* tests. In these analyses, the outcome variables were tested in turn, with the effects of both covariates and higher priority outcome variables removed. In the present study, this technique was deemed more appropriate than standard univariate *F* tests, as it allowed for the adjustment of the (likely) conceptual and statistical relatedness between the outcome variables [23]. Lastly, post hoc comparisons were employed to gain further insights into the between-group effects.

Alpha was set at the conventional level of 0.05 for all analyses. Differences between the three conditions were expressed in partial η^2^, considering effect sizes of 0.01 as small, 0.06 as medium, and sizes of 0.14 as large effects, respectively.

## Results

### Preliminary analysis

Prior to main analyses, the means, standard deviations, and correlations of the main constructs were examined. As predicted, students’ perceived overall gains were positively correlated with experience in statistical, writing, information retrieval, and critical judgment skills. Additionally, gender appeared to be negatively associated with information retrieval skills, statistical skills, and overall gains, suggesting that female medical students perceived themselves as being less experienced in these fields. Age was only positively correlated to information retrieval skills, and no associations between GPA and any of the EBM and research skills were found. Table 2 displays the zero-order correlations and summary statistics for the outcome variables and suggested covariates.

**Table 2:** Means, standard deviations, and correlations between main constructs

	1	2	3	4	5	6	7	8
1. Gender	1.00							
2. Age	−0.16	1.00						
3. GPA	−0.10	0.00	1.00					
4. Information retrieval skills	−0.17*	0.22**	0.03	1.00				
5. Writing skills	−0.01	0.14	0.14	0.49**	1.00			
6. Statistical skills	−0.30**	0.10	0.13	0.44**	0.52**	1.00		
7. Critical judgment	−0.03	−0.13	0.16	0.10	0.20*	0.25**	1.00	
8. Overall gains	−0.19*	0.11	0.07	0.50**	0.33**	0.49**	0.18*	1.00
Mean	–	22.23	7.01	2.45	2.55	1.95	3.05	3.52
Standard deviation	–	2.41	0.72	0.73	0.70	0.70	0.71	0.55

Mean scores for evidence-based medicine skills range from 1 (no experience) to 5 (much experience)

Gender: 0 male, 1 female, *GPA* grade point average

* *p* < 0.05, ** *p* < 0.001

To examine the power of the three covariates to adjust students’ perceived skills, a preliminary series of MANCOVA was run. In line with correlations, the overall *F* values revealed a statistically significant overall effect of gender (*F*(5, 134) = 4.24, *p* = 0.001), but not of age (*F*(5, 134) = 1.95, *p* = 0.09), or GPA (*F*(5, 134) = 1.36, *p* = 0.25) on the five outcome variables. Interaction effects among covariates and various skills were non-significant as well, indicating that student group membership did not vary with any of student background characteristics. Given these results, only gender was controlled for in the remaining analyses.

### Main analysis

Using Wilks’ criterion, the omnibus test revealed a statistically significant main effect for group membership, *F*(10,286) = 4.48, *p* < 0.001, partial η^2^ = 0.14 (90 % CI [0.05, 0.17]), suggesting multivariate group differences on the set of EBM skills. Gender appeared to only provide significant adjustment for statistical skills (*β* = −0.26, *p* < 0.01) and overall gains (*β* = −0.20, *p* < 0.05). To determine the nature of the main effect of group membership on students’ skills, a Roy-Bargmann stepdown analysis was performed, in which adjustment was made for both gender, and outcome variables. This analysis evaluated writing skills in step one, followed by information retrieval skills, statistical skills, critical judgment skills and overall gains in the second to sixth step, with higher priority variables treated as covariates. Results are shown in Table 3.

**Table 3:** Univariate F, stepdown F, and effect size for evidence-based medicine and research skills

Dependent variable	Univariate *F*	df	Stepdown *F*	df	Partial η^2^	90 % CI	
						Lower	Upper
Writing skills	11.11**	2/147	11.11**	2/147	0.13	0.05	0.21
Information retrieval skills	13.32**	2/147	5.40**	2/146	0.07	0.01	0.14
Statistical skills	2.31	2/147	0.53	2/145	0.01	0.00	0.04
Critical judgment	1.19	2/147	3.23*	2/144	0.04	0.00	0.10
Overall gains	1.12	2/147	2.02	2/143	0.03	0.00	0.08

*Df* degrees of freedom, *CI* confidence interval

* *p* < 0.05, ** *p* < 0.001

After adjusting for differences in gender, the three groups differed significantly in their perceived experience in writing, stepdown *F*(2, 147) = 11.11, *p* < 0.001, partial η^2^ = 0.13 (90 % CI [0.05, 0.21]). Unique contributions to explaining information retrieval skills, stepdown *F*(2, 146) = 5.40, *p* < 0.05, partial η^2^ = 0.07 (90 % CI [0.01, 0.14.]), and critical judgment, stepdown *F*(2, 144) = 3.23, *p* < 0.05, partial η^2^ = 0.04 (90 % CI [0.00, 0.10]) were found as well. No between-group differences were found for statistical skills and overall gains.

### Post-hoc analysis

Post-hoc contrasts showed that students in the undergraduate research project identified themselves as having significantly more experience in writing (*M*_*adj*_ = 20.63, *SE* = 0.72) than those in the basic skills elective (*M*_*adj*_ = 17.66, *SE* = 0.64, *p* < 0.01) and lecture-based skills course (*M*_*adj*_ = 16.15, *SE* = 0.62, *p* < 0.001). Significant mean differences between the perceived writing skills of groups 2 (basic skills elective) and 3 (lecture-based skills course) were not evident. Additionally, group 1 (undergraduate research project) had significantly more self-perceived experience in retrieving information (*M*_*adj*_ = 21.88, *SE* = 0.79) than the two other groups (group 2: *M*_*adj*_ = 19.06, *SE* = 0.67, *p* < 0.01; group 3: *M*_*adj*_ = 18.55, *SE* = 0.66, *p* < 0.01), with non-significant differences between the information retrieval skills of the two groups. Lastly, after adjustment of the higher priority outcome variables and the covariate, only groups 1 (undergraduate research project) and 3 (lecture-based skills course) differed significantly in their perceived experience in critical judgment (*M*_*adj*_ = 17.08, *SE* = 0.68 vs. *M*_*adj*_ = 19.41, *SE* = 0.56, *p* < 0.05).

## Discussion

This study is one of the first to compare students’ perceived EBM skill acquisition in several introductory skills courses in the undergraduate medical curriculum. Results showed that there were differences in students’ perceptions of having experience in various types of EBM skills in the three skills courses. Specifically, adjusted mean scores of students’ skill in writing and in retrieving information were significantly higher for students in the undergraduate research project than for students in the basic skills elective or the lecture-based course. This outcome was to be expected since undergraduate research projects, more than any other curriculum course, showcase the particular knowledge and skills for the kinds of academic thinking and writing as expected by scholarly journals [24, 25]. Lundgren and Halvarsson [26], for instance, noted that research projects enhance students’ abilities to carry out literature searches, and to systematically structure their reading materials. In addition, Löfgren and Ohlsson [27] found a positive effect of the experience of thesis writing on students’ preparedness for subsequent research projects.

Students in the authentic undergraduate research project did not perceive to have gained more experience in critical judgment skills than their peers in the other two courses. Despite the fact that students may take advantage of engaging in intellectual dialogues with their supervisors [28], it may be that students’ skills in critical judgment play an important role in other parts of the medical curriculum as well. During their block courses, for example, medical students continually explore different ways of thinking about a topic or disease, or critically judge the opinions of others. In addition to this, students perceive the research project as one of the most substantial and solitary activities of their studies [29]. Since these students often end up working at home on their projects, they may have less opportunities to discuss topics and scientific information than students who participate in the basic skills elective or, in particular, the lecture-based skills course. The idea that critical judgment may be a socially constructed activity rather than a solitary task might explain, in part, why the latter group of students differed from the undergraduate research project group in their perceived experience in critical judgment skills.

Students’ self-evaluations of their statistical skills appeared to be parallel in the three groups. It may be possible that students in the undergraduate research project chose to write an individual literature review, rather than a clinical or empirical project. In so doing, they did not necessarily require statistical data analysis techniques to finish their project and, consequently, did not feel they had made more progress in this skill than students who only briefly encountered statistical techniques during their lecture-based skills course. Therefore, investigators should take account of the type of project students undertake to gain further insights into students’ statistical skill acquisition.

Lastly, students who participated in authentic research activities such as the undergraduate research project or, to a lesser extent, the basic skills elective, did not seem to feel they had gained a larger amount of overall EBM skills, compared with students who took part in the traditional skills course. This lack of between-group differences suggests that these gains may be relatively well embedded in large-scale, lecture-based courses as well, at least in the perception of undergraduate medical students.

### Limitations

Several limitations of this study need to be considered. More specifically, the three groups were not randomized, as students self-selected the condition in which they participated by registering for the three (elective) skills courses in year 2 and 3. Due to the retrospective character of the study, it was also not possible to include pre-test measures of EBM skills in the research design. However, no discrepancies between groups in baseline characteristics (age, gender, and GPA) were found, nor did the adjustment for these covariates result in alterations in research findings.

Due to the nature and the design of this study, causal relationships between the variables of interest could not be drawn. Also, the timing of the courses in years 2 and 3 of the undergraduate curriculum prevented us from generalizing the results to the graduate years of medical school, where students are expected to demonstrate their EBM skills and abilities at a greater level of complexity. Longitudinal designs could deepen the understanding of how different skills courses affect medical students’ EBM skill acquisition at the end of their three-year Bachelor and beyond.

The response rate of this study could also be considered to be quite low (26 %). Given that the respondents in this study were essentially self-selected, this might possibly have resulted in response bias, and thereby, reduced the generalizability of the results.

Lastly, students’ self-perceptions were used to measure their EBM skill acquisition. Although self-report questionnaires may be advantageous in that they directly give participants’ own perception of their skill acquisition, their validity and reliability may at the same time be limited because such measurements are vulnerable for biases related to self-assessment [30]. This is backed up by the fact that female students stereotypically perceived themselves as being less experienced in statistical skills, and felt they made less progress than their male counterparts. Several investigators [21, 22] underscored the potential of the CSEQ for evaluating student activities that are well-related to objective measures of achievement. Still, using more objective measures, such as actual performance indicators or grouped self-assessments of learning outcomes [31], can significantly increase the confidence with which conclusions can be drawn from the data.

## Conclusion

In conclusion, authentic skills courses in the undergraduate medical curriculum appear to have several benefits over traditional lecture-based courses. Specifically, students who completed their undergraduate research project perceived themselves as more experienced in writing and information retrieval skills than students who participated in the other two skills courses. Students in the lecture-based skills course, however, identified themselves as being more experienced in critical judgment, suggesting that this skill is best learnt through dialogue. Also, students perceived their overall gains and experience in statistical skills to be equal across the three courses. Thus, in a time when medical schools are not always able to make meaningful research activities available as a part of the core curriculum, traditional, lecture-based skill courses do seem to be a reasonable alternative for at least part of the skills required for evidence-based research and practice in the undergraduate curriculum.

## Essentials

- Authentic research activities such as the undergraduate research project may, according to students, be more effective than traditional skills courses in equipping them with writing and information retrieval skills.
- Students perceive traditional skills courses to be more effective for acquiring critical judgment skills than authentic research activities.
- Critical judgment skills may be best learnt through dialogue with teachers and peers.

## Appendix

Subscales of the CSEQ (adapted from Kuh et al. [19]).

### Information retrieval skills

1. Used the medical library as a quiet place to read or study.
2. Found something interesting while browsing.
3. Asked a librarian/staff member for help with finding relevant information.
4. Read additional articles, next to assigned material.
5. Used index or database to find material.
6. Wrote bibliography/reference list for a term paper or other writing assignment.
7. Went back to read basic reference.
8. Made a judgment about the quality of information.

### Writing skills

1. Used a dictionary or thesaurus to look up the proper meaning of (medical) words.
2. Thought about grammar, sentence structure, word choice, and sequence of ideas or points as you were writing.
3. Asked other people to read something you wrote to see if it was clear to them.
4. Referred to a book or manual about writing style, grammar, etc.
5. Revised a paper or composition two or more times before you were satisfied with it.
6. Asked an instructor or staff member for advice and help to improve your writing.
7. Prepared a major written report for a block or course (20 pages or more).

### Statistical skills

1. Memorized formulas, definitions, technical terms, and concepts.
2. Used mathematical terms to express a set of relationships.
3. Explained your understanding of some scientific or mathematical theory, principle or concept to someone else (fellow student, co-worker, etc.).
4. Read articles about scientific or mathematical theories or concepts in addition to those assigned for a class.
5. Completed an experiment or project using scientific methods.
6. Practised to improve your statistical skills in using a piece of scientific equipment or statistical software.
7. Showed someone else how to use a piece of scientific equipment/statistical software.
8. Explained a statistical procedure to someone else.
9. Compared the scientific method with other methods for gaining knowledge and understanding.
10. Explained the empirical cycle to someone else.

### Overall gains

1. Acquiring knowledge and skills applicable to your future job as a clinician.
2. Acquiring knowledge and skills to conduct evidence-based medicine and research.
3. Gaining a broad general education about different fields of knowledge.
4. Gaining a range of information that may be relevant to your future career.
5. Broadening your acquaintance with the structure and quality of scientific literature.
6. Developing the ability to conduct a literature study.
7. Seeing the importance of the history of medicine and understanding the present as well as the past.
8. Writing clearly and effectively.
9. Presenting ideas and information effectively when speaking to others.
10. Using computers and other information technologies.
11. Becoming aware of different cultures, philosophies, and ways of life.
12. Developing your own values and ethical standards with respect to medical practice.
13. Understanding yourself, your abilities, interests, and personality.
14. Developing the ability to get along with different kinds of people.
15. Developing the ability to function as a member of a team.
16. Developing knowledge about, and understanding of the nature of scientific methods and statistics.
17. Understanding new developments in medicine, science, and technology.
18. Becoming aware of the (societal) consequences of new scientific applications.
19. Thinking critically and analytically.
20. Analyzing quantitative problems (understanding proportions, probabilities, etc.).
21. Putting ideas together, seeing relationships, similarities, and differences between ideas.
22. Learning on your own, pursuing your ideas, and finding the information you need.
23. Learning to adapt to change in the medical field.
